# So Long and Thanks for All the Fish: Overexploitation of the Regionally Endemic Galapagos Grouper *Mycteroperca olfax* (Jenyns, 1840)

**DOI:** 10.1371/journal.pone.0165167

**Published:** 2016-10-25

**Authors:** Paolo Usseglio, Alan M. Friedlander, Haruko Koike, Johanna Zimmerhackel, Anna Schuhbauer, Tyler Eddy, Pelayo Salinas-de-León

**Affiliations:** 1 Fisheries Ecology Research Lab, University of Hawai‘i, Honolulu, Hawai‘i, United States of America; 2 Fundación In-nova Castilla la Mancha, Madrid, España; 3 Pristine Seas, National Geographic Society, Washington, DC, United States of America; 4 Department of Marine Science, Charles Darwin Research Foundation, Santa Cruz, Galapagos Islands, Ecuador; 5 University of British Columbia, Vancouver, British Columbia, Canada; 6 Department of Biology, Dalhousie University, Halifax, Canada; California Polytechnic State University, UNITED STATES

## Abstract

The regionally endemic Galapagos Grouper, locally known as bacalao, is one of the most highly prized finfish species within the Galapagos Marine Reserve (GMR). Concerns of overfishing, coupled with a lack of fishing regulations aimed at this species raises concerns about the current population health. We assessed changes in population health over a 30-year period using three simple indicators: (1) percentage of fish below reproductive size (L_m_); (2) percentage of fish within the optimum length interval (L_opt_); and (3) percentage of mega-spawners in the catch. Over the assessed period, none of the indicators reached values associated with healthy populations, with all indicators declining over time. Furthermore, the most recent landings data show that the vast majority of the bacalao caught (95.7%,) were below L_m_, the number of fish within the L_opt_ interval was extremely low (4.7%), and there were virtually no mega-spawners (0.2%). Bacalao fully recruit to the fishery 15 cm below the size at which 50% of the population matures. The Spawning Potential Ratio is currently 5% of potential unfished fecundity, strongly suggesting severe overfishing. Our results suggest the need for bacalao-specific management regulations that should include minimum (65 cm TL) and maximum (78 cm TL) landing sizes, slot limits (64–78 cm TL), as well as a closed season during spawning from October to January. It is recognized that these regulations are harsh and will certainly have negative impacts on the livelihoods of fishers in the short term, however, continued inaction will likely result in a collapse of this economically and culturally valuable species. Alternative sources of income should be developed in parallel with the establishment of fishing regulations to limit the socio-economic disruption to the fishing community during the transition to a more sustainable management regime.

## Introduction

Overexploitation of numerous fisheries stocks is occurring worldwide [1],while the Galapagos Archipelago is often thought as a veritable Eden teeming with charismatic fauna, it has been far from spared by overfishing, as exemplified by the sea cucumber and lobster fisheries [2–4].

Fisheries in the Galapagos started as early as the 18^th^ century with the targeting of marine mammals [5]. In the mid 1920s, a group of Norwegian settlers came to the Galapagos and established a fish canning industry that ultimately proved to be unsuccessful [6]. Prompted mainly by the profitability of the sea cucumber and lobster fisheries, the artisanal fishing sector in the Galapagos grew dramatically by 325% to almost 1000 fishers between the years 1971 and 2000 [7]. This growth in the fishing sector was paralleled by immigration from mainland Ecuador, resulting in a rapid population increase from 1,500 people in the 1950s, to over 25,000 in 2010 [8]. Overfishing of both sea cucumbers and lobsters led to a decrease in the number of fishers since the year 2000, to an estimate of ~400 active fishers in 2012 [9,10].

Today finfish fisheries in the Galapagos target 68 different species [9], one of the most iconic of which is the Galapagos Sailfin Grouper (*Mycteroperca olfax*, locally called bacalao). Since the beginning of the fishery, in the early 1920s bacalao has been salted and consumed during the Easter holiday in a traditional dish called “fanesca” [6], giving the species both a high cultural and economic value. Yet, due to its restricted range and evidence of fisheries declines, bacalao has been listed as Vulnerable (VU) by the International Union for Conservation of Nature (IUCN) [11]. Fishing for bacalao occurs from three distinct groups of vessels: pangas (3.8–17.5 m long wooden hull); fibras (5 to 9.6 m long fiberglass hull); and botes (larger wooden boats 8–17.5 m long) [12]. Regardless of the vessel, bacalao is fished mostly with hook and line (locally called empate) [6], with few fishers still using (illegal) spearguns. Nowadays, the fishery is carried out as either single day trips from pangas or fibras that target islands and locations close to the fishing ports, or multiday trips where botes tow a number of smaller fibras or pangas and usually target locations farther from the fishing ports [13]. This results in differences in landings with virtually all of the landings from fibras and pangas coming from the central zones, while landings from botes reach 57% from the island of Isabela (mainly western zone), 14.8% from the islands of the northern zone (Darwin and Wolf), and the remaining from islands in the central zones [13]. The bacalao fishery is a year-round fishery that peaks during the months of October to April [6,13].

While bacalao fishing in the archipelago started in the 1920s, fishery research didn’t start until the 1960s. A concerted effort to collect fisheries data in the late 1970s and early 1980s resulted in the first in-depth description of the bacalao fishery [6,14]. This collection effort was interrupted in the 1980s with a decrease in governmental funding resulting from a fall in global oil prices [5]. Due to the exponential growth of the fishing sector, the Charles Darwin Foundation (CDF) started the Galapagos Fishery Monitoring Program, which later became the Participatory Fisheries Monitoring Program (PIMPP). This program collected spatially-explicit finfish fishery data from 1997 to 2006 [5]. However, after that time, data were only collected in 2009 by the work of von Gagern [12]. Because of this, a temporally continuous record of landing data for the bacalao fishery does not exist.

Despite the importance of this resource, there has been evidence of population declines. Early stock assessments determined bacalao harvest to be at sustainable levels [6,15], yet, Usseglio et al. (2015)[16] showed that bacalao grow slower, live longer, and mature at a greater age than previously assumed, which makes this species much more susceptible to overfishing than formerly thought. While bacalao comprised nearly all of the finfish landings in 1940s, it made up < 17% in recent years [6,17]. Reconstruction of fisheries landings in the Galapagos shows that groupers as a whole declined sharply in the late 1980s [17]. Older fishers perceptions agree with the decline of bacalao population, however, younger fishers did not perceive similar declines, reflecting shifting baselines in perception [18].

Under the current co-management scenario, fishers are represented as part of the Participatory Management Board (JMP- Junta de Manejo Participativo), where management proposals are agreed by consensus and then elevated to a national body for ratification [19]. While this co-management approach has reached important milestones, such as the management plan for the Galapagos Marine Reserve, the zonation of the marine reserve, and the fishing calendar; Ten years after its inception the optimal use of marine resources in the Galapagos has yet to be achieved [9]. Weak governance, that has lead to overfishing, emerges as the central problem facing the optimal use of marine resources in the Galapagos [9]. The main fishing regulation tools for finfishes in the management plan are the zonation scheme that delimits fishing and no-fishing areas, a licensing system, gear restrictions (e.g. spearfishing or long-lining), a ban on industrial fishing vessels (> 18 m centerline length), and a ban on the capture, transport and marketing of sharks [5]. Aside from sharks, there are no specific management regulations for bacalao or any other fish species for which there is a fishery in the Galapagos, being these only available for two species of high value invertebrates: a sea cucumber (*Isostichopus fuscus*) and three lobster species (*Panulirus gracillis*, *P*. *penicillatus*, *Scyllarides astori*)[19].

The lack of continuous fisheries time series data is common in most developing countries, and has led to the development of an array of length-based methods that are used to better understand the dynamics of data-limited fisheries [20,21]. Because of the high commercial, cultural, and ecological importance of bacalao, this paper has two main objectives: (i) compile the most comprehensive landing length dataset on bacalao to obtain a historical perspective of the fishery; and (ii) collect updated information to assess the current state of the fisheries. Both of these objectives will then provide information that will be used to produce suggestions for the effective management of this important fishery.

## Materials and Methods

### Ethics statement

The Galapagos National Park Directorate (GNPD) granted permissions PC-19-11, PC-24-13, and PC-25-14 approving this research, animal use approval was granted by the Animal Care & Use Committee, University of Hawai‘i, under protocol number 11–1284.

### Study area

**Fig 1:**
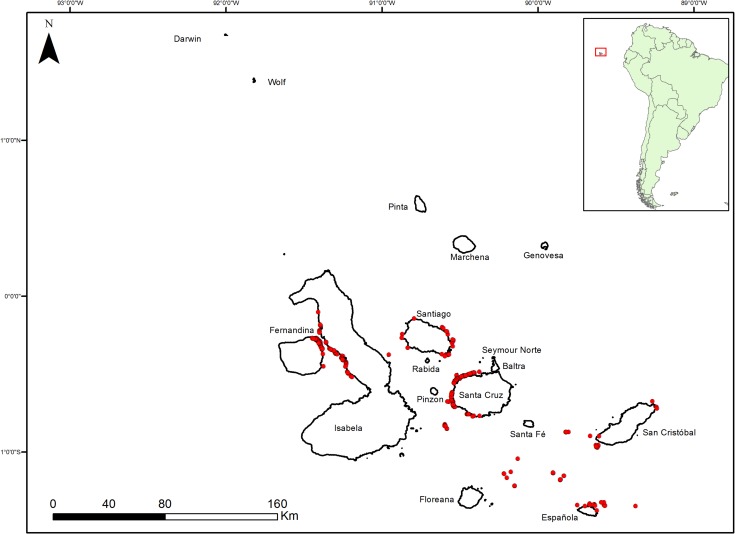
Study area of the Galapagos Archipelago, inset map showing location of the Galapagos Archipelago relative to South America. Red circles represent fishing locations for the 2012 sampling period.

Encompassing an area of approximately 133,000 km^2^, the Galapagos Marine Reserve (GMR) lies 1,000 km off the coast of mainland Ecuador. It was the first marine reserve established in Ecuador, and in 2001 was inscribed as a UNESCO World Heritage Site [22]. Extensive descriptions of the archipelago’s climate and oceanography can be found in [23]. Harris (1969) [24] proposed a division of the archipelago into five distinct hydro geographic zones based on physical characteristics. Further research has suggested broad-scale geographic patterns across the archipelago, based of faunal abundance and species richness, that divide the archipelago into three main regions: 1) the far northern islands of Darwin and Wolf, 2) the central, southern and eastern islands (including the east coast of Isabela) and 3) a western zone composed of the island of Fernandina and the western side of the island of Isabela [25] (Fig 1).

Bacalao is distributed in all areas of the archipelago, albeit with higher abundances in the colder central regions than the northern zone [13]. However, these differences were not found to be significant when comparing the abundance of bacalao among the 13 main islands of the archipelago [13]. The extent to which each of the data sources used in our analysis covers each of the zones is discussed below.

### Data sources

In order to assemble the most complete record of landing length of bacalao, we used the following datasets:

#### Historical photographs

Since no data from the early days of the fishery exists, and in order to obtain a historical perspective from this period, we followed the approach proposed by McClenachan (2009) [26] and sourced historical photographs of bacalao landings from archival collections. We obtained a total of eight photographs from the 1925–1938 period: the sources were the Rolf Blomberg (1934) (n = 1) and Allan Hancock (1938) (n = 6) expeditions to the Galapagos from their archives in Quito (Blomberg) and University of Southern California (Hancock), and an additional photo from a book by Hoff (1985) [27].

#### 1983

All raw data from Reck (1983) was lost in a fire at the CDF in 1984 [12], therefore length frequency data was obtained by extrapolating from the digitized time series published by Reck (1983) [6] using the software GraphClick 3.0.3. This dataset includes data collected from the islands of the central zone, as well as the western side of Isabela, and was collected during the fishing season running from October to March, between the years 1978–1981. Data were lacking from the northern zone.

#### 1998–2003 (PIMMP database)

The Galapagos Fishery Monitoring Program (Programa de Investigacion y Monitoreo Pesquero Participativo) ran from 1997–2006. It used fishery observers who recorded landings during the period of April to March (of the following year) at the fishing ports of Santa Cruz, San Cristobal and Isabela [28–30]. Landing data included the islands in the central and western islands. It is, however, not clear to what extent the monitoring covered the northern zone in each of these years. From this dataset we were only able to obtain data for the years 1998–2003.

#### 2009

Data for this period came from the work done by von Gagern (2009) [12], who recorded bacalao landing size at the Port of Puerto Ayora during the period October 2008 to March 2009, covering several islands in the central zone and the western area.

#### 2011

Data was collected by recording the length of bacalao landed at the fishing port of Puerto Ayora, on the island of Santa Cruz, for the period November 2010 to January 2011 and August-November 2011. Sampling covered landings fished on different islands within the central zone, lacking data for the western and northern zones.

#### 2012

This latest dataset was collected by accompanying fishers on their fishing trips during the periods of April-June, September-December in 2012, and January-February 2013, at several locations around the central and western zones (Fig 1). Detailed descriptions of the sampling methodology can be found in [16,31]. However, due to logistics constraints, data for the January-February 2013 period covered only the island of Santa Cruz. Length distributions were obtained from this dataset.

Across all data sources, except historical photographs, the main data collection method remained constant, as landings were assessed at fishing ports or on board fishing vessels and every fish was measured with a tape measure. From the data available it is not possible to assess the error associated with the measuring device, but it is assumed as constant across all methods.

### Data Analysis

#### Analysis of archival photographs

Archival images were analyzed to obtain estimates of bacalao landed length. We therefore selected images where fish were located in the same plane of an object of known length in order to perform length estimations on the same planar surface as described by [32]. This approach follows photogrammetric principles to be able to measure objects on a plane under perspective projection [33,34]. We obtained three separate length estimations for each fish under three separate original planes to minimize the error associated with these measurements and obtained a mean estimate length for individual fish. All analyses were conducted using the vanishing point tool in Adobe Photoshop CS4.

While this method yielded an estimate of mean length of bacalao in the early days of the fishery, it must be stressed that these data are subject to multiple biases (see discussion) that prevents it from being used in all further analyses.

#### Historical perspective of the fishery

Because the distinct data sources that we used lacked either a spatial component, or accurate data on the year’s fishing effort or landings, it was not possible to employ traditional fishery indicators, such as catch per unit effort (CPUE), to gain an understanding of the behavior of the fishery over time. As the common element in all datasets was the collection of size at landing data, we used the framework proposed by Froese (2004) [35], which relies on three simple indicators that are estimated from size at landing and reflect the health of the fishery. These indicators are: 1) percentage of mature fish in the catch (L_m_), 2) percentage of fish caught at optimum length (L_opt_) (defined as the length where the maximum yield and revenue can be obtained from a fish), and 3) percentage of mega-spawners (oldest and largest fish) in the catch [35].

The indicators were calculated as follows: 1) L_m_: Size at first maturity for bacalao was recently estimated at 65.3 cm TL [16]. 2) L_opt_: optimum length at catch was estimated using the empirical relationships between optimum length and ultimate maximum length (L_∞_) derived from the equation (L_opt_ = 10 ^1.0421*log^_10_^(L^_∞_^)-0.2742^ [36], which was then used to estimate the optimum length interval as ± 10% of L_opt_ [35]. Lastly, 3) mega-spawners are fish > L_opt_+10% [35].

Under Froese’s framework, in a healthy stock it would be expected that: (1) all fish caught are larger than the size at first maturity (a large percentage of the catch below size at maturity suggests recruitment overfishing); (2) a very large percentage of the catch within the optimum length interval (the inverse would suggest growth overfishing); and (3) values of percentage of mega-spawners in the landings to be ~ 30–40% in a population where there are no regulations regarding maximum landing size [35], such as is the case for bacalao in the GMR. In addition to these indicators we looked at mean length in catch, as it has been shown to be inversely correlated with fishing mortality [37]. Hence, a healthy fishery would be one where mean length at catch does not drop over time.

Changes in the percentage of fish in the landings above L_m_, within the L_opt_ interval, and percent mega-spawners were assessed by means of separate generalized linear models (GLM), assuming binomial variance structure. Percentage of each category, out of the total number of fish sampled per year, was the response variable for each GLM. Models were validated by graphical inspection of residuals showing that a linear effect of year was appropriate.

Changes in mean landing TL of bacalao over time was assessed by means of a mixed model GLM, where mean landed TL was modeled following a normal distribution, and modeling the source of each dataset as a random component to account for variability among data sources. The full model followed the formula TL~year+(1|data source). The models were fit using maximum likelihood and model fit was assessed by visual inspection of the residuals. Hypothesis testing was done by parametric bootstrapping (PB) over 1000 iterations. These analyses were conducted using the statistical software R version 3.0.2 [38], and the statistical packages lme4 [39] and pbkrtest [40].

#### Current fishery assessment

As our length composition data for 2012 represents the most recent dataset describing the bacalao fishery, we used a series of length-based methods to calculate indicators to assess the bacalao stock status that could then be used to provide management advice. We followed the framework proposed by Hordyk et al (2014) [20] to assess Spawning Potential Ratio (SPR), and fishery selectivity. We then assessed mortality and annual survival through a catch curve analysis. And lastly we assessed generational turnover time.

#### Spawning Potential Ratio (SPR)

SPR is derived by estimating the actual average lifetime production of mature eggs per recruit (P) at an equilibrium population density in the absence of any density-dependent suppression of maturation or fecundity [41]. It was estimated following the methods outlined in Hordyk et al. (2014)[20], which require length composition data, the ratio between natural morality (M) and Brody growth coefficient (*k*) M/*k*, asymptotic length (L_∞_), and the coefficient of variation of L_∞_(CV L_∞_), SPR is then calculated as:

$$SPR=\frac{P_{fished}}{P_{unfished}}$$

Where P is potential fecundity, and:

$$P_{fished}=\sum_{a}\{\begin{array}{c} E_{a}\;for\;a=0\\ e^{-Z_{a}-1^{a}1}E_{a}\;for\;0<a\leq a_{max} \end{array}$$

$$P_{unfished}=\sum_{a}E_{a}e^{-M_{a}}$$

where *a* is age, *E* is egg production at age *a*, *Z*_*a*_ is total mortality at age *a*, and *M*_*a*_ is maturity at age *a*.

While an estimate for *k* exists for bacalao [16], no such estimate exists for an unfished population of bacalao. In order to obtain an estimate for the M/*k* parameter which is needed as an input to the model, we used an estimate of *m* from a congener (*Mycterperca venenosa*) *m* = 0.29 [42]. Simulation results from Prince and colleagues [21], indicate predictable patterns in life-history ratios and the M/*k* parameter. Using our best knowledge of bacalao life history, we considered that our estimate of M/*k* = 2.7 corresponds to simulation results from [21] for fish with indeterminate growth and relatively late stage reproduction.

#### Fishery selectivity

A selectivity curve was modeled following the methods of Hordyk et al. (2014) [20], assuming that selectivity was asymptotic and size dependent following the equation:

$$S_{l}=\frac{1}{1+e^{-ln(l-l_{s50})/l_{s95}-l_{s50}}}$$

Where *S*_*l*_ is selectivity at length *l*, *l*_*s95*_ and *l*_*s50*_ are the lengths at 50 and 95% selectivity.

#### Mortality

Instantaneous total mortality (Z) was estimated using catch curve analysis following the methods outlined by Chapman and Robson (1960) [43]. An age frequency distribution for bacalao landings was constructed and catch plotted against age. For catch curve analysis, only the ages on the descending limb of the curve following peak catch were included, so as to only include size or age classes that are fully recruited to the fishery [44]. The Chapman-Robson estimate of annual survival (S) is given by the equation:

$$S=\frac{T}{1-T-\frac{1}{n}}$$

where n is the total recorded age of fish on the descending limb of the catch curve, and *T* is the sum of re-coded ages of fish on the descending limb of the curve. An estimate of Z is obtained following the equation proposed by [45]:

$$Z=-\log\left(S\right)-\frac{\left(n-1)(n-2\right)}{n(T+1)(N+T-1)}$$

Total mortality at age was modeled following the methods of [20] following the equation:

$$Z_{a}=M+S_{a}+F$$

where *Z*_a_ is total mortality at age *a*, M is the instantaneous rate of annual natural mortality, S_*a*_ is the selectivity at age *a*, and *F* is the annual instantaneous rate of fishing mortality.

The above analysis was conducted using the R package FSA [46].

#### Generational turnover rate

We estimated the average time required for a new generation to replace the last generation by calculating the mean generational turnover rate ($\bar{GT}$) following the methods proposed by [47] and applied to fishes by Depczynski and Bellwood (2006) [48], as given by the equation:

$$\bar{GT}=AM+\frac{T_{max}-AM}{2}$$

where *AM* is age at maturity in females, and *T*_*max*_ is maximum age, both of these estimates were obtained from [16].

## Results

### Historical perspective of the fishery

#### Historical photographs

**Fig 2:**
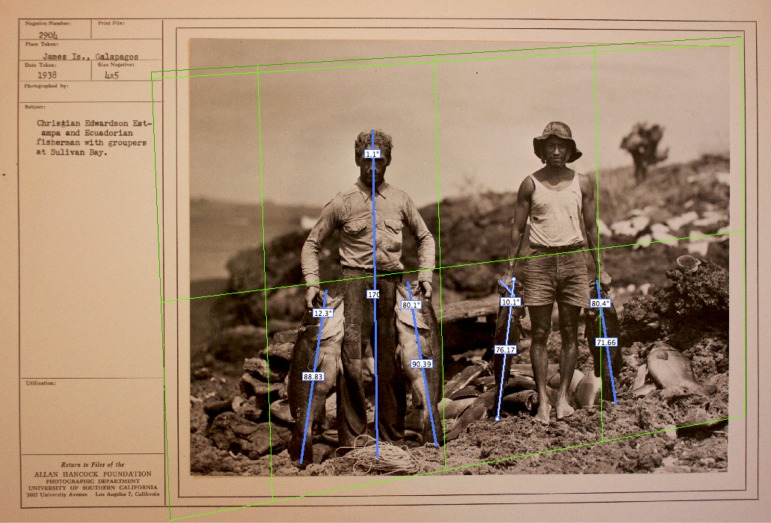
Historical photograph from 1938 showing a typical fishing trip for bacalao, with grid and measurements used in our analysis. Photograph courtesy of the University of Southern California, on behalf of USC Libraries.

A total of 25 bacalao were measured from the eight sourced photographs, an example photograph from 1938 shows 4 measured fish (Fig 2).

Overall mean of the 25 measured fish was 76.6 cm (± 15.5 sd) (Table 1), however, biases associated with the use of these historical photographs (see discussion) preclude the use of this data in any further analysis.

**Table 1:** Estimated size of 25 bacalao landed in the Galapagos from analysis of eight historical photographs from the years 1925, 1934 and 1938.

Photo	Source	Year	Fish #	Estimated TL (cm)
1	Stein Hoff	1925	1	65.3
			2	76.0
			3	76.3
2	R. Blomberg	1934	4	60.3
			5	112.6
			6	82.7
3	R. Blomberg	1934	7	61.4
4	R. Blomberg	1934	8	62.8
			9	43.5
5	R. Blomberg	1934	10	74.2
			11	115.0
			12	83.2
6	R. Blomberg	1934	13	70.1
7	R. Blomberg	1934	14	70.4
			15	69.2
			16	78.6
			17	86.3
			18	77.0
			19	61.1
			20	74.4
8	A. Hancock	1938	21	87.8
			22	91.7
			23	76.7
			24	71.0
			25	87.1

Estimated TL (cm) of bacalao from historical photographs from 1925 (n = 3), 1934 (n = 17) and 1938 (n = 5).

#### Assembled length landing dataset

The assembled dataset included a total of 5100 measured bacalao. Table 2 shows the number of fish sampled per data source, sampling effort expressed as the number of fishing trips sampled, and the mean Tl of bacalao landed. Inputs to calculate the fishery indicators were as follows: the reported maximum length for bacalao was 120 cm TL [49], length at maturity has been estimated at 65.3 cm TL, and L_∞_ = 110 [16]. Based on these values, L_opt_ was calculated as 71.3 cm TL (60.3–84.3 se). The L_opt_ interval was 64.2 to 78.4 cm TL, and mega-spawners were determined to be those fish > 78.4 cm TL, S1 File.

**Table 2:** Indicators to assess bacalao fishery over time.

Year	Sampled fish	Number of fishing trips sampled	L_m_	L_opt_	Mega-spawners	Mean landed TL cm (sd)
1983	1299	89	17.2	12.4	4.8	54.7 (11.5)
1998	544	116	14.5	13.2	2.9	53.4 (12.3)
1999	160	131	20	17.5	2.5	52.5 (13.4)
2000	264	25	11	11.4	0.8	49.6 (11.4)
2001	553	NA*	18.4	15.2	5.4	53.2 (13.4)
2002	292	104	11.6	11	1.7	49.8 (13.3)
2003	191	315	13.1	16.2	0.5	53.3 (10.2)
2009	663	NA*	12.8	11.8	3.3	53 (11.6)
2011	314	51	10.5	10.5	1.9	52.2 (10.7)
2012	489	45	4.3	4.7	0.2	46.8 (8.8)

Indicators used to evaluate bacalao fishery in the Galapagos: percent of fish above reproductive maturity (L_m_), percent of fish within the optimum length interval (L_opt_), percent mega-spawners, and mean landed TL. For bacalao landed in the Galapapos during the years 1983, 1998–2013, 2011, and 2012.

* No metadata on this dataset was available, S2 File.

**Fig 3:**
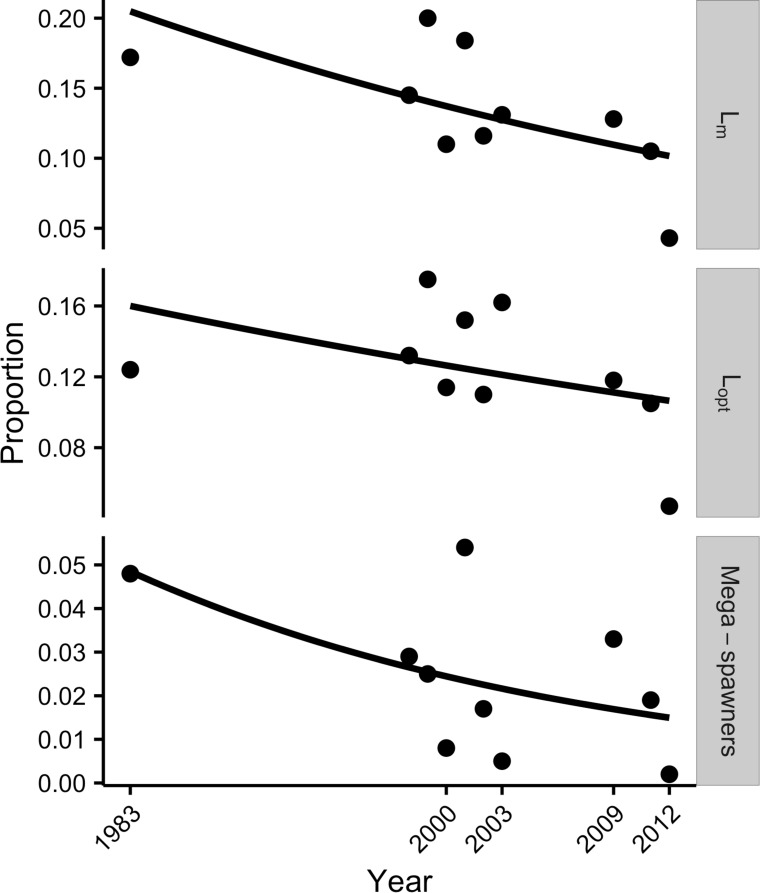
Binomial GLMs to assess changes overtime in the three indicators used to evaluate stock health: percent of fish above reproductive maturity (L_m_), percent of fish within the optimum length interval (L_opt_), and percent mega-spawners. For bacalao landed in the Galapapos during the years 1983, 1998–2003,2009, 2011, and 2012.

The three indicators used to assess the fishery (L_m_, L_opt_, and mega-spawners) showed declines over time with significant differences among years L_m_ (χ^2^ = 6.54, p<0.05), L_opt_ (χ^2^ = 35.02, p<0.05), and mega-spawners (χ^2^ = 20.04, p<0.01) (Table 2 and Fig 3). Furthermore, the minimum values expected for healthy populations were not met in any of the years.

**Fig 4:**
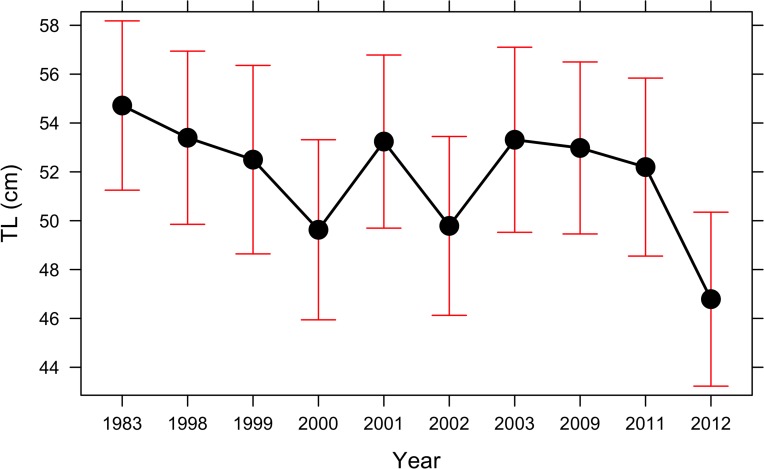
Generalized linear mixed model estimates of mean TL(cm) for bacalao caught in the Galapagos during the years 1983, 1998–2003, 2009, 2011 and 2012 (error bars ± SE).

Mean TL of landed bacalao showed a significant decline over time, (PB test = 81.5, p<0.01) with a decrease in size of 7.9 cm (3.6 se) in the 30-year period (Table 1 and Fig 4).

### Current fishery assessment

Samples collected by fishing in 2012 resulted in a total of 489 bacalao from the islands of Isabela, Santa Cruz, and Santiago, with an overall mean TL of 46.8 cm (± 8.8 sd), and a range from 20 to 79 cm TL.

#### SPR

Using an M/*k* ratio of 2.7, the length-based estimate of bacalao SPR for the year of 2012 was 0.05 (±0.008 SD) that is 5% of an unfished stock. The F/M ratio was estimated as 1.98 (±0.2 SD), suggesting a very high fishing mortality.

#### Fishery selectivity

**Fig 5:**
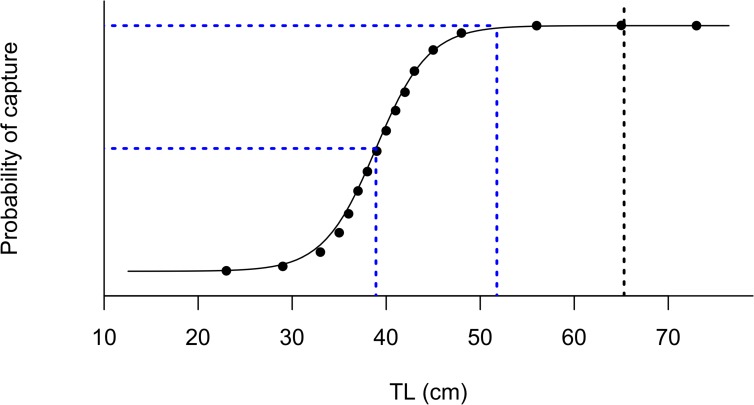
Selectivity of bacalao from length data collected in 2012. The black line represents the logistic selectivity model, the blue lines represent size at 50% and 100% recruitment to the fishery, and the black dotted line represents size at which 50% of the population reaches sexual maturity (Lm).

The size at which 50% of the bacalao recruit to the fishery was estimated at 39 (±0.73 SD) cm TL and 95% of fish are recruited at 46.7 (±1.37 SD) (Fig 5).

#### Mortality

Annual survival (S) was estimated at 45.3 (2.3 se), and total mortality (Z) at 0.79 (0.04 se).

#### Generational turnover time

Using an estimate of age at first maturity of 6.5 years, and a maximum observed age of 21 years [16], the generational turnover time for bacalao was 13.7 years.

## Discussion

While the bacalao artisanal fishery in the Galapagos has occurred for over 90 years, the lack of a comprehensive record of landings makes its assessment an arduous task. In this paper we used a series of indicators based on landing size (e.g., TL, L_m_, L_opt_, percentage of mega-spawners) to infer population status of bacalao. Results from this evaluation show declining trends of these indicators over the last 30 years. Moreover, these indicators were not met in the most recent dataset from 2012, suggesting both recruitment and growth overfishing. In recent landings, the percentage of fish above size at maturity has dropped to 4%, and a very low SPR (5%) suggest that the bacalao stock in the Galapagos is in danger of collapse due to reproductive failure. Currently the GMR has no management regulations specific to bacalao, relying solely on no-take areas that have not shown positive results for bacalao [13]. Our results highlight the urgent need to establish management regulations specific to this species in the Galapagos.

We recognize that there are sources of error associated with datasets that are collected by a variety of methods and people over the years, we have tried to acknowledge these biases whenever possible.

### Historical perspective of the fishery

Our results show that the three metrics employed to assess the health of the bacalao population (L_m_, L_opt_, and mega-spawners) were below expected levels across all years analyzed, and that there had been a significant decline over the 30-year period analyzed. Similarly, mean landed TL of bacalao also showed significant declines over time. These results suggest that, under the framework proposed by [35], the bacalao population shows evidence of both recruitment and growth overfishing.

As data from the early days of the fishery does not exist, we looked into archival images to assess landing size of bacalao in those early years. While archival images have been successfully used to assess changes in trophy fish [26], it must be recognized that this method is prone to bias as archival images might be biased towards large “trophy” individuals only, or purposely exclude them. Unfortunately, no metadata associated with the images we employed allows us to discern either of these biases. Furthermore, the limited number of images, the small sample size, and the unknown spatial coverage limits the credibility of this estimate on its own. Nonetheless, it is important to note that even if these images are biased towards trophy fish, the largest fish in our catch in 2012 was of only 79 cm TL, with one 100 cm TL individual reported by [16] in comparison to a 115 cm TL estimate from the historical photographs. Our results from the 30-year analysis suggest that mean landed size has significantly changed since the 80s, making it very likely that individuals of bacalao that were landed in the early days of the fishery were larger.

### Recruitment overfishing

Recruitment overfishing, or diminishing the ability of the population to replenish itself [35], is evident when a large proportion of the landings are below size at maturity, and is further compounded by the removal of large individuals from the population given their disproportionate contribution to the reproductive success of a population [50,51]. Our results suggest that on average, every year during the last 30 years, roughly 80% of the bacalao population in the main fishing areas in the central and western zones have been fished before reproducing.

Large and old individuals (i.e. mega-spawners) should represent 30–40% of the age structure in a healthy population where there are no regulations of maximum landing size [35] which is the case for bacalao in the Galapagos. The proportion of mega-spawners across years was very low, suggesting that overfishing of bacalao may have started in the early days of the fishery and probably even before data collection began in the 1980s. Having so few large individuals greatly reduces the reproductive potential of the population. Given that fecundity increases exponentially with female weight [52], smaller individuals have reduced reproductive potential [53], and egg size and early survival of larvae is reduced in young females [54]. In a fishery were a disproportionate amount of large individuals has been removed the resulting size and age distribution will be truncated towards younger and smaller spawners [50,51,54], which can in turn lead to what is dubbed “longevity overfishing” [55].

### Growth overfishing

The proportion of fish within the optimal capture interval for the study period ranged between 10–20%, suggesting that bacalao is captured before attaining a weight that would ensure maximum revenue. While this represents a substantial economic problem, it has also been noted that growth overfishing can be more important than recruitment overfishing in increasing the risk of stock collapse [56]. Selectivity at catch is often used to mitigate this form of overfishing [56,57]. However, this method poses an interesting problem for the bacalao fishery as the fishing method employed has very low selectivity resulting in capture of individuals across the size range of the species with the same gear [31].

### Changes in landed TL

It has been shown in multiple overexploited stocks that the mean size of the landings will decline when larger individuals are removed from a population [58–60]. Our results show that the mean landed size of bacalao has decreased considerably over time to its lowest point in 2012. It is important to note, however, that the 2012 dataset might be biased against larger individuals, as only the island of Santa Cruz was sampled during the reproductive season. Bacalao is fished throughout the archipelago, with fishers favoring locations in the central and western zones, and only 14% of the landings coming from the northern islands [13]. Our data sets for all years cover the central and western zones, where over 80% of the landings occur, with a lack of coverage from the northern islands (Darwin and Wolf) in the most recent datasets (2011–2012). Nicolaides (2002) [13] did not find differences in the abundance of bacalao among the 13 main islands in the archipelago. A recent discovery of a bacalao spawning aggregation in the northern islands revealed a mean observed TL of 71 cm at the aggregation site [61]. While differentiation of landings from different zones goes beyond the scope of this paper, it is acknowledged that landings from the northern islands may bias the results from the most recent datasets as individuals there might be larger.

Some groupers are known to be territorial with limited range overlap where older, larger individuals migrate to spawning sites [62,63], this could bias sampling done in different seasons as larger individuals might not be present. Our data sources, nonetheless, covered the reproductive period for bacalao which peaks during the months of October-April [14,64]. As metadata for some of our data sources was not available, it was not possible to asses the sampling effort allocated to this period, however as most of the fishing effort directed at bacalao is done during the months of October to April [13], and descriptions of the data sources imply data collection in this period we assumed that all data sources covered the reproductive period. The extent to this coverage is, nonetheless, lower in the most recent dataset, as the reproductive period was only covered for the island of Santa Cruz. Yet the decreasing trend in landed TL was still significant even when removing the 2012 dataset from the analysis.

### Current assessment

Data for this assessment mainly covers the central and western zones, noting that the reproductive season was only sampled in the island of Santa Cruz. Our results might therefore not truly represent the fishery across the entire archipelago, but the central zone only. With this in mind, our results suggest that the assessed population can be considered in critical condition due to: 1) an SPR of only 5%, 2) size of recruitment to the fishery ~15 cm smaller than size at maturity, 3) very high total mortality and low annual survival, and 4) high fishing mortality as evidenced by the F/M ratio of 1.98.

An SPR of lower than 40% is considered risk adverse for many species, and for certain stocks it means overfishing [65]. Our results suggest that the Galapagos population of bacalao is at risk, and most probably overfished. Similarly, our results suggest that there is a 100% chance of catching immature individuals as fish are recruiting to the fishery ~15 cm below size of sexual maturity, which can be reflected in the severely skewed sex ratio observed [6,16,64]. In addition, early recruitment to the fishery leads to a reduction in the probability that females will survive to sex change [66], loss of productivity due to sperm limitation [67,68], and ultimately reproductive failure as males become too rare to effectively mate with females (Allee effect) [67,69]. The very low observed annual survivorship coupled with the very large proportion of immature individuals in the catch can have detrimental implications to the reproductive success of the population as discussed above. Lastly, the very high F/M ratio of 1.98 suggests a very high fishing mortality that is far greater that the reference point of F_MSY_ = 0.87M considered reasonable for teleosts [70]. While high values of the F/M ratio might be sustainable in highly selective fisheries that target only a few of the of the oldest year classes in a stock ([20]), spawning per recruit has been suggested to decrease drastically if the fishery catches a large proportion of immature individuals [20,71,72], as is the case of bacalao where the percentage of the catch above size of maturity has been consistently low ranging between 26 and 5% in the last 30 years. Even with the data limitations previously discussed, the above results are consistent with the historical perspective of older fishers suggesting that the resource is under extreme pressure [18].

At present there are no management regulations aimed specifically at bacalao in the GMR. Although there have been management proposals in the past [15], these have been largely ignored, placing present day protection of bacalao solely within the GMR zonation scheme, which has not shown to be effective for bacalao [13,73]. The bacalao fishery in Galapagos should follow conservative management approaches that include a mix of controls over catch and fishing effort, seasonal closures, and effective no-take spatial closures. It is relevant to note that the estimated population doubling time for bacalao, 14 years, implies that in a stable population this is the time taken to establish a new generation (i.e. two larvae surviving to adulthood and replacing two adults) [48]. This figure can be taken as a proxy for effects of protection to become noticeable, while these have been shown to occur between 4–7 years for groupers [60,74]. In the case of bacalao, with a longevity greater than 20 years [16], this might take longer.

### The way forward

The GMR is no stranger to fisheries collapses, as exemplified by the sea cucumber fishery, with efforts to implement regulations resulting in riots and violence [75]. Such happenings can be traced to a lack of credibility and legitimacy that fishers perceive in the co-management of the GMR [10,76], which in turn results in the observed pattern of lack of enforcement and high rates of noncompliance [77]. Our results strongly suggest that the bacalao fishery is experiencing both recruitment and growth overfishing. While a total closure until the stock recovers would be highly advisable, we recognize that this may not be feasible, as it will negatively impact the livelihood of a large number of fishers and their families. The challenge facing the GNPD is to balance regulations that would adequately protect bacalao, while ensuring that these will be followed. It is important to note the futility of setting “paper regulations” that will not be followed. There is an obvious need to further the participation of fishers in the decision making process to a fully cooperative approach where fishers are included in all of the research phases, as fishers note this as the major reason for their distrust in current management regulations [10].

We therefore, suggest that bacalao-specific management regulations be developed in conjunction with fishers, in an effort to produce a series of regulations that will improve the health of the fish stocks and the fishery and be likely to be accepted and followed by the fishing community. At a minimum, these regulations should be focused on addressing the main issues identified in this paper:

- Minimum landing size of bacalao ≥ L_50_ (65 cm TL) (recruitment overfishing)
- Maximum landing size of bacalao ≤ to size of mega-spawners (78 cm TL) (low numbers of mega-spawners).
- Closed season from October to January during peak spawning.
- Slot limits ~64–78 cm TL (growth overfishing)

In addition to the above regulations, it would be advisable to re-evaluate the existing no-take areas within the GMR in terms of essential fish habitat for bacalao. The final site selection of the conservation zones (no-take zones) in the GMR was mostly based on expert opinion, with the main focus of protecting a range of sites representative of different shallow habitats in each of the five biogeographic zones within the GMR [78]. However, there was limited use of biological data, compared to information based on tourism or fisheries [78]. This suggests a need to incorporate biological information such as juvenile nursery habitats, spawning aggregation sites, and essential fish habitat for adults in the design of no-take zones specific for bacalao. Positive work has already started with the recent declaration of a no-take area around the northern islands of Darwin and Wolf, which has potential to enhance fisheries given the fact that there is as least one known spawning aggregation for bacalao within the new MPA [61]

It is understandable that enacting these regulations will have negative impacts on the livelihoods of fishers from the GMR, emphasizing the need of working with fishers to develop alternative sources of income in parallel with the development of fishing regulations. This approach is plausible since > 60% of fishers recently interviewed in the GMR responded that they would be willing to adopt income alternatives different than fishing [10]. It is essential to recognize the choices facing the conservation, fishing, and community-at-large of the Galapagos Archipelago between managing their resources wisely or avoiding short-term political and social conflict. The former would ensure resilience of an important cultural, economic, and biological resource, while the latter would result in what Pauly aptly named “Malthusian overfishing” [79].

## Supporting Information

S1 File Raw data. Raw data for all analyses conducted in this paper.
(CSV)

S2 File Indicators. Fishery health indicator dataset.
(CSV)
